# Disparities in Cardiovascular Risk Factors in Northern Plains American Indians Undergoing Coronary Artery Bypass Grafting

**DOI:** 10.1089/heq.2018.0021

**Published:** 2018-08-01

**Authors:** Eric Anderson, Matthew Glogoza, Aaron Bettenhausen, Rory Guenther, Dylan Dangerfield, Rick Jansen, Roxanne Newman, Donald Warne, Cornelius Dyke

**Affiliations:** ^1^ University of North Dakota School of Medicine and Health Sciences, Grand Forks, North Dakota.; ^2^ Department of Cardiothoracic Surgery, University of Texas Health Science Center San Antonio, San Antonio, Texas.; ^3^ Department of Public Health, North Dakota State University, Fargo, North Dakota.; ^4^ Department of Cardiothoracic Surgery, Sanford Health Fargo, Fargo, North Dakota.

**Keywords:** cardiovascular health, diabetes, health disparities, minority health, coronary artery bypass graft

## Abstract

**Objectives:** Heart disease is the leading cause of death in American Indians (AIs). For AI patients with severe coronary artery disease requiring coronary artery bypass graft (CABG) surgery, little data exist. The purpose of this study was to evaluate short-term outcomes of Northern Plains AI undergoing CABG and identify variations in patient presentation.

**Methods:** All patients undergoing isolated CABG between June 2012 and June 2017 were studied. Seventy-four AI and 1236 non-American Indian (non-AI) patients were identified. Risk factors, preoperative characteristics, cardiac status, procedural information, and outcomes were collected. Univariate analysis comparing short-term clinical outcomes between AI and non-AI populations was performed. Multivariable logistic regression models were constructed and outcome differences assessed. Unadjusted Kaplan–Meier survival estimates were produced using 5-year survival data.

**Results:** AI patients presented with increased risk factors, including higher rates of diabetes mellitus (AI 63.5% vs. non-AI 38.7% *p*=< 0.001) and smoking/tobacco use (AI 60.8% vs. non-AI 20.0% *p*=> 0.001). Seventy-nine percent of AI patients resided on or near federal reservations and presented from rural locations. Internal mammary artery (IMA) graft use in both groups was high (AI 95.9% vs. non-AI 94.9% *p*=0.904), and multiarterial grafting with left internal mammary artery and radial artery use was common in both groups (AI 67.6% vs. non-AI 69.6% *p*=0.814). No significant differences in unadjusted 30-day mortality or short-term outcomes were detected. Adjusted Kaplan–Meier survival curves were similar between race groups up through 5 years after CABG (*p*-value=0.38).

**Conclusion:** AIs presented with significantly more risk factors for cardiovascular disease compared with the general population, with especially high rates of insulin-dependent diabetes and active tobacco use. Despite this, outcomes were similar between groups. In propensity-matched groups, AIs were at decreased risk for prolonged length of stay and combined morbidity/mortality. In contrast to previous reports, AI racial identity did not adversely affect survival up to 5 years after CABG.

## Introduction

Heart disease is the leading cause of American Indian (AI) deaths for all ages.^1–3^ AI individuals suffer heart disease-related deaths at younger ages when compared with U.S. whites as well as with all U.S. races.^3^ The incidence of cardiovascular risk factors such as diabetes and smoking is also increased in AIs.^4^ Within the AI population itself, significant variation in heart disease mortality exists with the highest mortality rates primarily located in South and North Dakota, Wisconsin, and Michigan.^5^ For patients with severe coronary artery disease needing coronary artery bypass grafting (CABG), ethnic differences in outcomes have been reported, although there is a paucity of data regarding CABG in AI patients.^6–11^ In an older retrospective study, an in-patient mortality of 4.5% in AIs was reported, significantly higher than for other ethnic groups.^12^

No study evaluating AI patients based on the Society of Thoracic Surgeons (STS) isolated CABG risk factors, STS short-term outcomes, or intermediate survival exists. In addition, it is unclear if AI race itself is a risk factor for poor outcomes as risk-adjusted outcomes after CABG have not been reported. Studies evaluating outcomes in AI patients are difficult as only 1.2% of the population self-identifies as AI.^13^ AIs, however, are the largest minority group in both North and South Dakota, making up 5.5% and 8.9% of each state's population, respectively.^13^ As a cardiac surgery referral center in North Dakota in the largest rural healthcare system in the Northern Plains, our institution has a unique opportunity to examine outcomes after CABG in this high-risk population. The purpose of our study was two-fold: to understand how AI patients present and identify potential variations in preoperative risk factors for CABG as well as evaluate short-term, perioperative outcomes following CABG in this vulnerable population.

## Methods

**FIG. 1.**
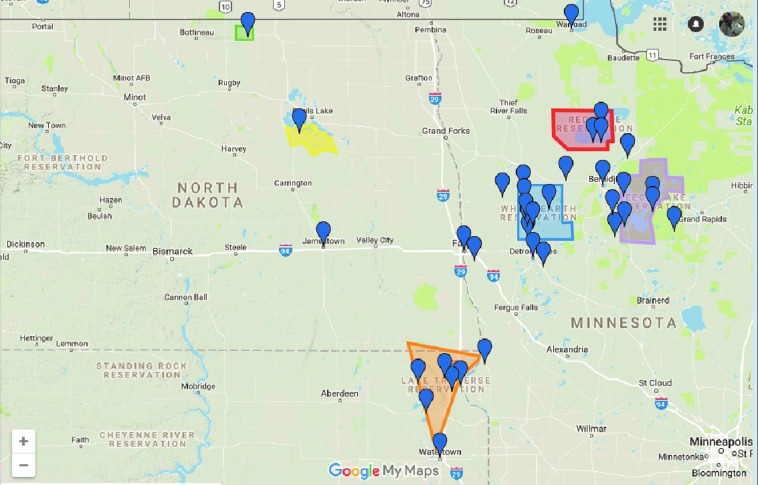
Geographic distribution of American Indian patients. The geographic distribution of American Indian patients and federally recognized reservation land are illustrated. All patients are represented but each pin may represent more than one patient.

Institutional Review Board approval was obtained from Sanford Health and the University of North Dakota School of Medicine and Health Sciences before conducting this study. Our referral area of ∼750,000 people includes eastern North Dakota, northern Minnesota, and northeastern South Dakota, as well as multiple Indian Reservation lands. Data for this single institutional study were collected prospectively but analyzed retrospectively. All isolated CABG patients from June 1, 2012 to June 1, 2017 were included. AIs were identified via Indian Health Services (IHS) referral or patient self-identification and resided both on and off recognized Reservation land (Fig. 1). Indications for surgery and procedural plan were determined by the surgical team, but the guiding operative philosophy within the group emphasized complete revascularization and multiple arterial grafting. Clinical risk factors, preoperative cardiac status, procedural information, and clinical outcomes as identified and defined by the STS Adult Cardiac Surgery Database for isolated CABG were collected for all patients. STS risk scores and the Universal Definition of Perioperative Bleeding (UDPB) score were calculated for all patients.^14^ All discharged patients were followed for 6 weeks after surgery in the cardiac surgery clinic, and further follow-up was obtained through the electronic medical record. Outcomes for all patients were documented, and there were no missing or undocumented patients. Operative mortality was defined as either occurring during the hospitalization, in which the operation was performed (even if after 30 days), or those deaths occurring after discharge from the hospital, but within 30 days of the procedure. STS-defined short-term outcomes included permanent stroke, prolonged ventilation, deep sternal wound infection (DSWI), renal failure, reoperation for any reason, major morbidity or mortality, short stay, and long stay.

### Statistical analysis

All data were analyzed using R statistical program (version 3.3.2). We tested the null hypothesis that there were no differences between AI and non-American Indian (non-AI) population for each preoperative risk factor and postoperative outcome. Significance of differences between AI and non-AI groups for categorical variables was tested using chi-squared, and continuous variables were tested using the non-parametric Wilcoxon Rank Sum test. Univariate and multivariable logistic regression analysis comparing nine short-term clinical outcomes between AI and non-AI populations was performed to test our null hypothesis. The univariate analysis allowed us to detect differences for a given outcome between AI and non-AI groups and the multivariable analysis was used to detect differences for a given outcome after adjusting for a specific set of clinical factors. Adjustment factors included those identified as significantly different (*p*-value <0.05 between AI and non-AI groups: gender [male/female], diabetes [Yes/No], diabetes control [None/Diet or Oral/Insulin/Other], smoker [Ever/Never], and age [continuous]). Kaplan–Meier survival estimate curves assessed 5-year survival differences following CABG between AI and non-AI groups using the Log Rank test. We also repeated the regression and survival analysis comparing AI to non-AI patients using a sample matched on STS Operative Mortality Risk Score (a tool incorporating multiple clinical risk factors for CABG) in an attempt to equalize baseline risks between the race groups and control for potential confounding risk factors. The propensity matching resamples the non-AI group matching on a list of specific variables (in this case: gender, diabetes, diabetes control, smoker, and age) and was done using R package MatchIt and which provides AI and non-AI groups which are comparable on the list of matched variables (gender, diabetes, diabetes control, smoker, and age). Statistical significance for all tests was predetermined at a *p*-value ≤0.05.

## Results

The AI population in this study was Northern Plains Ogalala Sioux, and the non-AI population was predominantly white. A small number of other races were classified as non-AI: African American 2 (0.2%), Hispanic 8 (0.6%), Asian 7 (0.5%), and Pacific Islander 1 (0.1%). The geographic distribution of AI patients and federally recognized reservation land are illustrated in Figure 1. Patient demographics and clinical presentation are presented in Table 1. Upon presentation to the hospital, significant differences in cardiovascular risk factors were found between AI (*n*=74; 5.7%) and non-AI (*n*=1236; 94.3%) patients. AIs as a group were younger and had a higher percentage of female subjects. AI subjects had higher rates of diabetes mellitus (AI 63.5% vs. non-AI 38.7% *p*=< 0.001) and were more likely to require insulin treatment of their diabetes mellitus (AI 36.5% vs. non-AI 17.2% *p*=< 0.001). Although not significant, there was a trend that AI patients were more likely to present with preexisting end-stage renal disease on hemodialysis and to present with preexisting peripheral vascular disease. Rates of smoking and tobacco use in the AI population were strikingly higher compared with the non-AI population (AI 60.8% vs. non-AI 20.0% *p*=< 0.001).

**Table 1.** **Preoperative Characteristics**

Variable^a^	AI% (*n*=74)	Non-AI% (*n*=1236)	*p*^b^
Age (years)	60.4±11.0	67.0±9.7	<0.001
Gender (Female)	36.5 (27)	20.4 (252)	0.002
Height (cm)	173.6±9.4	171.5±10.0	0.071
Weight (kg)	93.1±19.3	93.3±20.8	0.711
BMI	30.8±5.6	31.6±6.1	0.252
BMI category			0.511
Underweight/normal (<24.9)	10.8 (8)	12.9 (160)	
Overweight (25.0–29.9)	36.5 (27)	35.6 (441)	
Obese I (30.0–34.9)	25.7 (19)	31.0 (383)	
Obese II (35–39.9)	20.3 (15)	13.4 (166)	
Morbid obesity (40.0 +)	6.8 (5)	7.0 (86)	
Diabetes	63.5 (47)	38.7 (478)	<0.001
Diabetes control			<0.001
None/diet	45.9 (34)	65.6 (811)	
Oral	17.6 (13)	17.1 (212)	
Insulin	36.5 (27)	17.2 (213)	
Dialysis	5.4 (4)	1.8 (22)	0.081
HTN	89.2 (66)	80.9 (1000)	0.104
Chronic lung disease			0.260
No/mild	62.1 (46)	71.0 (877)	
Moderate	4.1 (3)	5.7 (71)	
Severe	8.1 (6)	4.4 (54)	
Smoker	60.8 (45)	20.0 (247)	<0.001
Immunosuppressed	6.8 (5)	4.7 (58)	0.599
PVD	18.9 (14)	11.6 (143)	0.088
CVD	8.1 (6)	10.4 (128)	0.673
Pre-op CHF	13.5 (10)	14.3 (177)	0.952
Previous PCI			0.100
No	66.2 (49)	76.2 (942)	
≤ 6 h	0.0 (0)	0.57 (7)	
>6 h	33.8 (25)	23.2 (287)	
Previous open heart surgery	1.4 (1)	3.6 (44)	NA
Previous CABG surgery	1.4 (1)	3.0 (37)	NA
Previous valve surgery	0 (0)	0.6 (7)	NA
Creatinine (mg/dL)	1.3±1.7	1.1±0.6	0.380
Preoperative hemoglobin	13.2±1.8	13.5±1.9	0.117
Status			0.829
Elective	35.1 (26)	33.0 (408)	
Urgent	54.1 (40)	57.5 (711)	
Emergent/emergent salvage	10.8 (8)	9.5 (117)	
Hx of MI			0.444
None	43.2 (32)	54.1 (669)	
<6 h	0.0 (0)	1.1 (13)	
6–24 h	6.8 (5)	5.2 (64)	
1–7 days	27.0 (20)	22.8 (282)	
>8 days	23.0 (17)	16.8 (208)	
Cardiac presentation			0.145
Stable angina	23.0 (17)	21.8 (270)	
Unstable angina	28.4 (21)	41.2 (509)	
Non-STEMI	39.2 (29)	29.9 (369)	
STEMI	9.5 (7)	7.1 (88)	
Arrhythmia			0.112
No	93.2 (69)	86.0 (1063)	
AF/AFL/other	6.8 (5)	14.0 (173)	
IABP			0.938
Yes	17.6 (13)	16.5 (204)	
Left main disease (>50%)	27.0 (20)	33.2 (410)	0.305

^a^ Categorical data are presented as % (number). Continuous data are presented as mean±standard deviation.

^b^ The chi-squared test was used to determine differences between race groups for categorical variables and the nonparametric Wilcoxon Rank Sum test was used to determine differences between race groups for continuous variables.

AF, atrial fibrillation; AFL, atrial flutter; AI, American Indian; BMI, body mass index; CABG, coronary artery bypass grafting; CHF, congestive heart failure; CVD, cerebral vascular disease; HTN, hypertension; Hx, history; IABP, intra-aortic balloon pump; MI, myocardial infarction; PCI, percutaneous coronary intervention; Pre-op, preoperatively; PVD, peripheral vascular disease; STEMI, ST-segment elevated myocardial infarction; NA, sample size too small calculate *p*-value.

Procedural details are found in Table 2. Approximately 80% of patients in both groups underwent complete revascularization with three or more grafts, and there was no difference in the number of grafts between groups. The incidence of internal mammary artery utilization was high in both groups, as was the incidence of two or more arterial grafts in both groups. There was no difference between the AI and non-AI groups with regard to complete arterial revascularization, and both groups had high rates of radial artery use as a conduit. There was no difference in off-pump CABG rates between the two groups (AI 54.1% vs. non-AI 53.0% *p*=0.954).

**Table 2.** **Operative Characteristics**

Variable^a^	AI% (*n*=74)	Non-AI% (*n*=1236)	*p*^b^
Number of grafts			0.199
One	4.1 (3)	4.0 (50)	
Two	13.5 (10)	20.8 (257)	
Three	52.7 (39)	50.1 (619)	
Four	20.3 (15)	20.3 (251)	
Five	9.5 (7)	3.6 (45)	
LIMA grafting	95.9 (71)	94.9 (1173)	0.904
Radial artery grafting	67.6 (50)	69.6 (860)	0.814
Complete arterial grafting	47.3 (32)	53.6 (662)	0.353
Off pump	54.1 (40)	53.0 (655)	0.954
UDPB classification			0.959
Zero	66.2 (49)	67.0 (828)	
One	12.2 (9)	11.5 (142)	
Two	14.9 (11)	13.0 (162)	
Three	6.8 (5)	8.0 (99)	
Four	0.0 (0)	0.40 (5)	

^a^ Categorical data are presented as % (number). Continuous data are presented as mean±standard deviation.

^b^ The chi-squared test was used to determine differences between race groups for categorical variables.

LIMA, left internal mammary artery; UDPB, Universal Definition of Perioperative Bleeding.

**FIG. 2.**
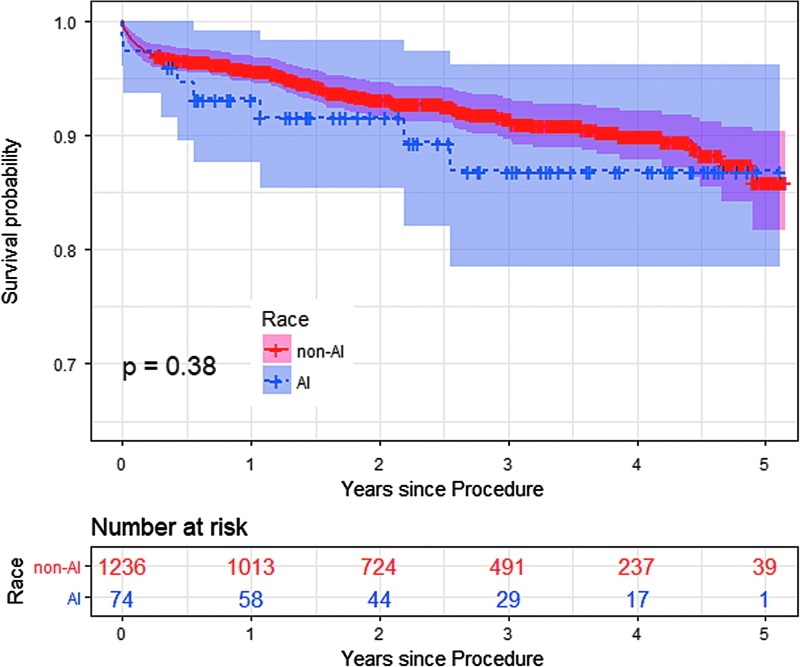
Survival estimates for all patients undergoing CABG. Kaplan–Meier survival estimates 5 years after CABG were not significantly different between American Indian and non-American Indian patients. CABG, coronary artery bypass graft.

There was no significant difference between the AI subjects and non-AI subjects for the unadjusted outcomes of operative mortality, permanent stroke, acute renal failure, prolonged ventilation, DSWI, reoperation, major morbidity or mortality, short stay, long stay, or average length of stay (Table 3). Risk-adjusted operative mortality and short-term outcomes based on multivariable logistic regression analysis adjusting for gender, diabetes, diabetes control, smoking, and age revealed no significant differences between the two race groups for the risk of the any of the nine outcomes (Table 3). The Kaplan–Meier 5-year survival curves were similar for AIs and non-AIs undergoing CABG after adjusted for STS scores (log rank *p*-value=0.38; Fig. 2).

**Table 3.** **Univariate and Multivariate Logistic Regression Outcomes Testing (AI=74, Non-AI=1236)**

		Unadjusted^*^				Adjusted^⋀^			
Outcome	No. of AI/non-AI patients	OR	Lower 95% CI	Upper 95% CI	*p*	OR	Lower 95% CI	Upper 95% CI	*p*
Operative mortality	2/20	1.69	0.27	5.94	0.486	1.26	0.19	4.98	0.771
Permanent stroke	2/8	4.26	0.64	17.40	0.070	2.30	0.31	11.27	0.343
Renal failure	0/24	NA	NA	3.45E+12	0.985	0.00	NA	8.01E+22	0.989
Prolonged ventilation—24 h	0/44	NA	3.58E-111	1.97E+08	0.984	NA	2.53E-107	2.07E+07	0.983
DSWI	0/9	NA	NA	7.50E+40	0.991	NA	NA	1.60E+37	0.990
Reoperation—any reason	2/54	0.57	0.09	1.89	0.448	0.48	0.08	1.69	0.334
Major morbidity or operative mortality	6/90	1.12	0.43	2.46	0.791	0.74	0.27	1.70	0.509
Short stay LOS—6 days	56/864	1.33	0.78	2.35	0.311	1.37	0.77	2.54	0.302
Long stay LOS—14 days	1/31	0.53	0.03	2.53	0.536	0.34	0.02	1.78	0.310

Univariate and multivariate logistic regression testing the association between race and nine different CABG-related outcomes. The ORs refer to odds that the outcome will occur in the AI population, relative to the non-AI population.

^*^ Unadjusted model only includes race (non-AI, AI).

^⋀^ Adjusted model includes: gender, diabetes (yes/no), diabetes control (none/diet/oral/insulin/other), smoker (ever/never), and age (continuous).

NA represents sample size too small to calculate OR.

Response variables for each individual model were operative mortality (yes/no) and all deaths occurring during the hospitalization. Permanent stroke (yes/no) any confirmed neurological deficit of abrupt onset. Renal failure (yes/no) increase of serum creatinine to >4.0 with an increase of at least 0.5–2.0 and 3×most recent preoperative Cr level. Prolonged ventilation (yes/no) ventilation >24 h. DSWI (yes/no) occurring within 30 days. Reoperation for bleeding, tamponade, or other cardiac or noncardiac reason (yes/no). Major morbidity or operative mortality (yes/no) composite endpoint defined as any of the outcomes listed above it in this table.

CI, confidence interval; DSWI, deep sternal wound infection; LOS, length of stay; OR, odds ratio.

**FIG. 3.**
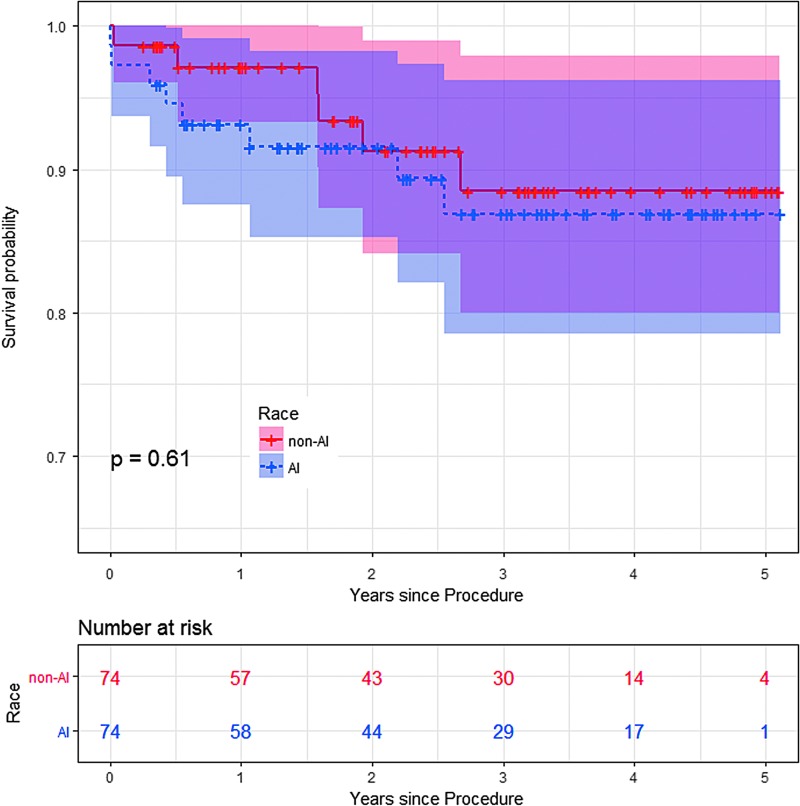
Survival estimates for the propensity-matched patients undergoing CABG. Kaplan–Meier survival estimates within the propensity-matched cohorts were not significantly different between American Indian and non-American Indian patients.

When propensity matching was used to select AI (*n*=74) and sample non-AI (*n*=74) patients based on comparable STS Operative Mortality Risk Score, none of the nine outcomes, including operative mortality, was observed to be significantly different in the unadjusted analysis (based on the significant cutoff of *p*-value <0.05; Table 4). After adjusting the logistic regression model for gender, diabetes, diabetes control, smoking, and age in this propensity-matched sample (Table 4), AIs were suggested to be less likely to have a prolonged length of stay (odds ratio [OR]=0.02; 95% confidence interval [CI]: 0–0.56; *p*-value=0.05) and were at a decreased risk for combined major morbidity/mortality and reoperation (OR=0.73; 95% CI: 0.28–0.92; *p*-value=0.05). Among this propensity-matched sample, the Kaplan–Meier 5-year survival curves were similar for AIs and non-AIs undergoing CABG (log rank *p*-value=0.61; Fig. 3).

**Table 4.** **Propensity Matched Sample Univariate and Multivariate Logistic Regression Outcomes Testing (*n*=148)**

	Unadjusted^*^				Adjusted^⋀^			
Outcome	OR	Lower 95% CI	Upper 95% CI	*p*	OR	Lower 95% CI	Upper 95% CI	*p*
Operative mortality	2.03	0.19	44.20	0.567	0.95	0.04	29.58	0.973
Permanent stroke	2.03	0.19	44.20	0.567	0.58	0.02	20.54	0.743
Renal failure	NA	NA	9.46E+238	0.996	NA	NA	Inf	0.998
Prolonged ventilation—24 h	NA	NA	Inf	0.997	NA	0.00E+00	Inf	0.999
DSWI	NA	NA	Inf	0.997	NA	0.00E+00	Inf	0.999
Reoperation—any reason	0.38	0.05	1.84	0.261	0.04	0.00	0.55	0.032
Major morbidity or operative mortality	0.64	0.20	1.87	0.417	0.21	0.04	0.92	0.049
Short stay LOS—6 days	0.80	0.37	1.75	0.584	0.73	0.28	1.93	0.526
Long stay LOS—14 days	0.32	0.02	2.56	0.328	0.02	0.00	0.56	0.053

Univariate and multivariate logistic regression testing the association between race and nine different CABG-related outcomes. The ORs refer to odds that the outcome will occur in the AI population, relative to the non-AI population.

^*^ Unadjusted model only includes race (non-AI, AI)

^⋀^ Adjusted model includes: gender, diabetes (yes/no), diabetes control (none/diet/oral/insulin/other), smoker (ever/never), and age (continuous)

NA represents sample size too small to calculate OR.

Response variables for each individual model were operative mortality (yes/no) all deaths occurring during the hospitalization. Permanent stroke (yes/no) any confirmed neurological deficit of abrupt onset. Renal failure (yes/no) increase of serum creatinine to >4.0 with an increase of at least 0.5–2.0 and 3×most recent preoperative Cr level. Prolonged ventilation (yes/no) >24 h. DSWI (yes/no) occurring within 30 days. Reoperation for bleeding, tamponade, or other cardiac or noncardiac reasons (yes/no). Major morbidity or operative mortality (yes/no) composite endpoint defined as any of the outcomes listed above it in this table.

## Discussion

Significant differences were present between the AI and non-AI populations undergoing CABG in the present study (Table 1). AIs were younger, more likely to be female, and had significantly more risk factors for coronary artery disease. The incidence of diabetes mellitus in the AI population was significantly higher than the general population, as was the number of diabetic patients requiring insulin. This may reflect a lack of resources for the AI population who may not have access to newer, non-insulin therapies for diabetes. It is unlikely due to obesity, as obesity rates as calculated using body mass index were similar between groups. Sixty percent of AIs presenting for CABG were active tobacco users, threefold higher than non-AIs (Table 1). In addition, although not statistically significant, there was a trend toward more patients with end-stage renal disease requiring dialysis in AIs, as well as a higher incidence of peripheral vascular disease. The high rates of cardiovascular risk factors in AIs in our study is consistent with previous reports.^1,5^ While there is considerable variability of tobacco use within AIs, the extremely high rate of smoking in our AI population is consistent with previous reports that Northern Plains Indians have the highest smoking rate of any AI region in the country.^5^ Among Northern Plains AIs, tobacco use is both ceremonial and secular, likely contributing to the extreme rates of tobacco exposure.

Over 75% of patients in both groups presented with acute coronary syndromes, and the rates of emergent or salvage surgery were similar between groups (Table 1). AI patients residing on or near reservation land were significantly removed from secondary or tertiary healthcare facilities and represent a challenge for expeditious management (Fig. 1). Despite this, there was no difference in rates of emergent surgery or higher acuity in AI patients. Other reports have noted that AI/Alaskan Native (AN) patients have the longest duration from symptom onset to arrival at a hospital compared to other ethnic groups and are less likely than non-Hispanic whites to undergo percutaneous coronary intervention or CABG.^15,16^ This difference from our study may be due to proximity of reservation territory to outlying hospitals as well as an infrastructure at our referral hospital allowing for air transport and rapid triage. It is conceivable that AIs in more remote locations may not have this advantage and this lack of healthcare access likely explains previously reported treatment delays.

Operative details between AIs and non-AIs were similar in our study (Table 2). Internal mammary artery utilization rates were high in both groups (∼95%). Surgical opinion in our center favors the use of multiple arterial grafting with the radial artery as the second (or more) arterial conduit, and the incidence of radial artery use was nearly 70% in both groups and was undeterred by high rates of smoking and peripheral vascular disease in AIs. While utilization of multiple arterial conduits is rising, our rate of radial artery use is nearly 10-fold higher than national norms using the STS National Database for comparison and reflects our preference for arterial revascularization (Table 2). Complete arterial revascularization was achieved in nearly 50% of patients using sequential arterial grafting techniques without the use of bilateral internal mammary arteries (BIMA). While it is recognized that BIMA use in diabetic patients can be safe,^17^ it is possible high rates of diabetes in the AI population influenced our preference for single internal mammary artery (IMA) graft use and radial grafting. We also speculate that the high incidence of multiple arterial conduits and the relatively high rate of complete arterial revascularization in this series may account for the similarity in survival in the short-term and at 5 years, despite increased long-term risk factors for cardiovascular disease.^18–21^

Using the STS risk score to adjust for potentially confounding preoperative variables, there were no differences in either unadjusted or risk-adjusted mortality between AIs and non-AIs, implying that AI race of itself was not a risk factor for mortality. This is in contradistinction with earlier reports, in which AI race was identified as an adverse risk factor.^12^ In fact, risk-adjusted multivariate logistic regression analysis of the propensity-matched sample suggests that AIs are less likely to have prolonged length of stay and have a lower combined risk of major morbidity/operative mortality or reoperation for any reason (“Adjusted” in Table 4). This suggests that when looking at AI and non-AI patients with equal baseline risk factors, AI patients may actually have better outcomes. Potentially, this could be a signal of greater resilience to risk factors in the AI population. This outcome will require validation with larger datasets as we acknowledge our analysis is limited by low event rates and small group sizes.

In contradistinction to previous reports, after risk adjustment, AI race was not a risk factor for operative mortality, stroke, bleeding score, or other complications in this study. Several potential explanations for this difference exist. In the Nallamothu et al.^12^ study, AIs were more than 10 times as likely to have their surgery done at a rural, community facility. Also, significant advances in surgical technique have been made since previous reports, including the use of multiple arterial conduits and improved medical and critical care. We believe that complete revascularization and multiple arterial conduits are particularly beneficial for patients with increased cardiovascular risk factors such as AIs. The similarity in operative mortality between AIs and non-AIs in our study is also consistent with published findings in Canadian Aboriginals undergoing a variety of cardiac surgical procedures, although data were limited to in-hospital mortality in that study.^22^ Finally, the possibility of a statistical error must be considered due to relatively small groups and low event rates. Potentially, a larger dataset of AI patients undergoing CABG may reveal disparities in outcomes undetected here.

Given the disparity of increased risk factors in AI patients requiring CABG, secondary prevention of coronary artery disease after CABG^23^ is critical for all patients, especially so for high-risk populations such as ours. Sood et al. have reported that Canadian Aboriginals were less likely to receive postcardiac surgery medications after cardiac surgery.^22^ Public health disparities also exist and may contribute to long-term disparities in outcomes after CABG beyond the 5-year intermediate survival outcomes in this study. Most of the AI population in our study resides on or near reservations and receives care through the IHS. In 2010, IHS federal funding was significantly below allocated federal spending per person in other healthcare programs,^24,25^ and less than a third of IHS healthcare providers report adequate access to specialty care hospitals, diagnostic imaging, and other services.^26^

The AI/AN population is also affected by social inequities, which may impact their health.^27^ The combination of personal cardiovascular risk factors, limited healthcare funding, geographic isolation, lack of access to specialty care, and social disadvantages has the potential to negatively affect long-term survival after CABG. While long-term follow-up and secondary prevention were not assessed as part of this study, they are potential areas where differences based upon race may occur and are an important area for further research.

## Limitations

There are several limitations to this study. This was a single-center, retrospective study. While the population of AIs undergoing CABG is relatively small, this is the largest single-site study to date of an AI population isolated CABG. Postoperative event rates overall were few, and STS risk score adjustment and propensity matching have inherent limitations. Significant regional differences and heterogeneity within the AI/AN population also exist, and therefore, our AI population of Northern Plains Indians may not be generalizable to other AI/AN regions or to the AI/AN population as a whole. The AI population in this study was also largely rural, with the vast majority of the patient coming from federal reservations and from a remote distance, and therefore, our results may not be generalizable to AI patients residing in major metropolitan areas, where access to healthcare may be more abundant or readily available. Finally, the possibility of unconscious treatment bias based upon race must be recognized, which may hinder, alter, or delay treatment.

## Conclusions

In this study, Northern Plains AIs undergoing CABG are younger, more likely to be female, and present with more severe risk factors for atherosclerotic disease with especially high rates of insulin-dependent diabetes and active tobacco use than a non-AI (generally white) population. Despite this, short-term perioperative morbidity and mortality rates were similar between groups and risk-adjusted survival estimates between groups were not significantly different up to 5 years after CABG. AI racial identity itself was not a risk factor for poor outcomes in contradistinction to previous reports. The signal of resilience in AIs with less combined morbidity after CABG will require further investigation. Northern Plains AIs may undergo CABG with acceptable and comparable survival to the general population.

## Abbreviations Used

AF: atrial fibrillation
AFL: atrial flutter
AN: Alaskan Native
AI: American Indian
BIMA: bilateral internal mammary arteries
BMI: body mass index
CABG: coronary artery bypass graft
CHF: congestive heart failure
CI: confidence interval
CVD: cerebral vascular disease
DSWI: deep sternal wound infection
HTN: hypertension
Hx: history
IABP: intra-aortic balloon pump
IHS: Indian Health Services
IMA: internal mammary artery
LIMA: left internal mammary artery;
LOS: length of stay
MI: myocardial infarction
non-AI: non-American Indian
OR: odds ratio
PCI: percutaneous coronary intervention
Pre-op: preoperatively
PVD: peripheral vascular disease
STEMI: ST-segment elevated myocardial infarction
STS: Society of Thoracic Surgeons
UDPB: Universal Definition of Perioperative Bleeding
